# Pilot study of a two-arm non-randomized controlled cluster trial of a psychosocial intervention to improve late life depression in socioeconomically deprived areas of São Paulo, Brazil (PROACTIVE): feasibility study of a psychosocial intervention for late life depression in São Paulo

**DOI:** 10.1186/s12889-019-7495-5

**Published:** 2019-08-22

**Authors:** Marcia Scazufca, Maria Clara P. de Paula Couto, Maiara Garcia Henrique, Ana Vilela Mendes, Alicia Matijasevich, Paula Carvalho Pereda, Renato M. Franzin, Antônio Carlos Seabra, Pepijn van de Ven, William Hollingworth, Tim J. Peters, Ricardo Araya

**Affiliations:** 1 LIM-23, Faculdade de Medicina, Instituto de Psiquiatria, Hospital das Clínicas (HCFMUSP), Universidade de São Paulo, São Paulo, Brazil; 2 Departamento de Medicina Preventiva, Faculdade de Medicina (FMUSP), Universidade de São Paulo, São Paulo, Brazil; 3 Departamento de Economia, Universidade de São Paulo, São Paulo, Brazil; 4 Departamento de Engenharia de Sistemas Eletrônicos, Universidade de São Paulo, São Paulo, Brazil; 5 Department of Electronics & Computer Engineering, University of Limerick, Limerick, Ireland; 6 Department of Population Health Sciences, Bristol Medical School, University of Bristol, Bristol, UK; 7 Centre of Global Mental Health, Institute of Psychiatry, Psychology, and Neurosciences,King’s College, London, UK

**Keywords:** Older adults, Depression, Pilot study, Primary care, Collaborative care

## Abstract

**Background:**

Depression is a common and recurrent condition among older adults and is associated with poor quality of life and increased health care utilization and costs. The purpose of this pilot study was to assess the feasibility of delivering a psychosocial intervention targeting depression, and to develop the procedures to conduct a cluster randomized controlled trial among older adults registered with primary care clinics in poor neighbourhoods of São Paulo, Brazil.

**Methods:**

We conducted a pilot study of a two-arm cluster, non-randomized controlled trial. Two primary care clinics adhering to the Family Health Strategy were allocated to either the intervention or the control arm. In the control arm, patients received enhanced usual care consisting of staff training for improved recognition and management of depression. In the intervention arm, alongside the enhanced usual care, patients received a 17-week psychosocial intervention delivered by health workers assisted with an application installed in a tablet.

**Results:**

We randomly selected 579 of 2020 older adults registered in the intervention clinic to participate in the study. Among these individuals, 353 were assessed for depression and 40 (11.0%) scored at least 10 on the PHQ-9 and were therefore invited to participate. The consent rate was 33/40 (82%) with a resulting yield of 33/579 (5.7%). In the control arm, we randomly selected 320 older adults among 1482 registered in the clinic, 223 were assessed for depression and 28 (12.6%) scored 10 or above on the PHQ-9. The consent rate was 25/28 (89%), with a resulting yield of 25/320 (7.8%). Of the 33 who consented in the intervention arm, 19 (59.4%) completed all sessions. The mean PHQ-9 at follow-up (approximately 30 weeks after inclusion) were 12.3 (*SD* = 3.7) and 3.8 (*SD* = 3.9) in the control and intervention arms, respectively. Follow-up rates were 92 and 94% in control and intervention arms, respectively.

**Conclusions:**

Identification and engagement of clinics, randomization, recruitment of individuals, measures, and baseline and follow-up assessments all proved to be feasible in primary care clinics in São Paulo, Brazil. Results support the development of a definitive cluster randomized controlled trial.

**Trial registration:**

This study was retrospectively registered with Registro Brasileiro de Ensaios Clínicos (ReBEC), number RBR-5nf6wd. Registered 06 August 2018.

## Background

Most Low-Middle-Income Countries (LMIC) are experiencing a rapid growth of their ageing populations. According to the latest population census, Brazil has approximately 20 million people aged over 60 years (11% of the population), most of whom live in poverty and isolation [1], and it is expected that there will be 73.5 million older adults in 2060. Depression is a common chronic condition among older adults [2–5] and is associated with poor quality of life [6, 7], adverse social and health events [8–10], and increased health care utilization and costs [11, 12].

Health care systems in LMIC are not well prepared to meet the mental health challenges associated with these population changes. Unsurprisingly, depression in later life often goes unrecognized and untreated [13–16]. A survey of older adults living in poor neighbourhoods in São Paulo, Brazil, found that less than 5% of cases of depression were identified by Family Health Teams (primary care services), and that among those identified with depression, only 12.3% were receiving treatment [17].

The most effective treatments for depression in later life have been developed and tested in high-income countries [18–20]. These are complex, multiple component interventions delivered in primary care, with several health workers simultaneously collaborating on delivering a care plan (collaborative model). Resources in these programmes are allocated according to the specific needs of the patient (hence, stepped-care model). Although there is evidence from high-income countries of effective treatments for depression in later life, generalizing from this evidence to LMIC is problematic given socio-cultural and health system differences. The World Health Organization recommends that the treatment of depression should be delivered predominantly in primary care [21, 22]. The integration of mental health care into primary care is still far from adequate in Brazil and most other LMIC, where successful depression programmes based on collaborative care models are hard to find.

Simple, feasible, and affordable primary care interventions aimed at treating depression in older adults are therefore needed in Brazil and other LMIC experiencing similar demographic transitions [23]. These interventions should target the main barriers to treat depression in these settings, such as: patients’ social isolation and mobility problems; health workers’ difficulty in identifying depressive symptoms and lack of skills and support needed to deliver effective interventions; poor coordination, continuity of care and accountability within health teams; scarcity of resources; and unavailability of specialized mental health care [13–16, 24]. Such interventions should be developed and piloted thoroughly before being subject to definitive evaluations.

We therefore developed and evaluated the feasibility of a collaborative care depression programme for depressed older adults with strong community-based and task-shifting [25] components customized to the existing Brazilian primary care setting. In this paper, we present the results of a two-arm, non-randomized controlled cluster study aimed at evaluating the feasibility of the intervention and of a future randomised controlled trial to test its effectiveness. The cluster design was chosen to avoid contamination, as community health workers responsible for delivering the psychosocial intervention could be employed across clusters. This pilot study provides an important opportunity to identify potential difficulties and challenges and the necessary refinements of our research procedures, before we conduct the definitive cluster randomized controlled trial (RCT) to investigate the cost-effectiveness of the collaborative care programme for late life depression.

### Aims

1. To assess the feasibility of recruitment, assessments and random selection of participants in each clinic.
2. To obtain an estimate of the variability of the outcome across clinics and recruitment/retention rates to inform sample size calculations for a definitive RCT.
3. To evaluate the feasibility of delivering the psychosocial intervention, and to compare the performance of various health workers delivering the intervention.
4. To assess the feasibility of collecting information on use of resources, including costs associated with intervention delivery, and health data from existing health system databases, to conduct an economic evaluation during the definitive trial.

## Methods

This is a two-arm pilot study of a non-randomized, controlled cluster trial.

### Study setting

The study was conducted in primary care clinics adhering to the Family Health Strategy (FHS) [26] in São Paulo, Brazil. Each clinic provides comprehensive and continuous care for inhabitants from a defined catchment area. Health professionals within the clinic work in Family Health Teams (FHT). Each team is responsible for up to 4000 inhabitants. In 2017, there were 42,105 FHTs deployed across the country providing health cover to 130,487,012 Brazilians, approximately 63% of the population [27]. Two clinics located in Northern São Paulo were invited to participate in the study. The managers of these clinics agreed participation in the pilot study. It was decided a priori that the first clinic to accept participation would become the intervention arm. This clinic had seven FHTs, whilst the control clinic had three FHTs. In both arms, the FHTs comprised a family doctor, one nurse, two NAs, and six CHWs. Both NAs and CHWs must have completed secondary education, but NAs need to successfully complete an additional nursing technical course of one-year duration. CHWs, on the other hand, learn their skills through hands-on experience and continuous education. CHWs are also required to be residents in the catchment area for that clinic.

### Participants

Eligible participants were individuals aged 60 years and older registered with the two participating clinics. The exclusion criteria were: Patient Health Questionnaire-9^28^ (PHQ-9) score < 10; complete deafness; terminal illness; risk of suicide; or an inability to communicate (e.g., due to cognitive impairment either reported by a family member or detected by the researcher). The exclusion criteria were checked by the research assistants during recruitment and baseline assessments.

### Assessments

#### Recruitment

All CHWs at the intervention and control clinics were asked to provide a list with all their patients aged 60 years or older. From those lists, a random sample of potential participants was selected for the recruitment interview, through computer generated numbers which were managed by a research assistant unaware of the clinic’s allocation. All interviews were carried out either by phone or home visits by trained research assistants blinded to participants’ allocation. All questionnaires were read out to participants. The research team tried to contact by phone each of the sampled participants three times. If phone calls were not successful after the third attempt, the research team made three attempts to visit the potential participant at home. During recruitment, information on participants’ education, income, and job status was gathered. The assessment of depression (primary outcome) was conducted with the PHQ-9 [28]. The PHQ-9 is a well-validated brief depression measure extensively used in primary care and clinical research in a large number of countries, including Brazil [29], which is sensitive to changes over time [30, 31]. The PHQ-9 comprises nine questions, each one rated from 0 (not at all) to 3 (all the time).

#### Baseline

All participants who scored at least 10 in the PHQ-9 (cut-off point for depression) at recruitment were approached for a face-to-face assessment at home. This assessment was carried out as soon as possible after recruitment. If the baseline assessment was performed more than 28 days after recruitment, the PHQ-9 was repeated. This procedure was needed for 42 participants, with 11 of them scoring < 10 in the second PHQ-9, resulting in the exclusion and replacement of these individuals. During the baseline assessment, the following information was gathered: 1) Sociodemographic information including gender, city/state/country of origin, race, marital status, socioeconomic status, religious activities. 2) General health status was assessed using self-reported history of hypertension, diabetes, cancer, and stroke. 3) Quality of life was assessed with the European Quality of Life 5 Dimensions-5 levels version (EQ-5D-5 L) [32]. 4) Capability of older people with the Investigating Choice Experiments for the Preferences of Older People-CAPability (ICECAP-O) [33, 34]. and physical disability (use of cane, wheel chair, diapers, and being bedridden). 5) Social support and stressful life events [35]. 6) Consumption of alcohol (Alcohol Use Disorders Identification Test - AUDIT) [36] and tobacco. The treatment started approximately 2 weeks after baseline assessment was carried out.

Written informed consent was gained from all participants for the recruitment and baseline interviews.

#### Follow-up

Follow-up assessment took place approximately 4 weeks after the end of the intervention (26 to 32 weeks after baseline PHQ-9). It consisted of a face-to-face interview at the participant’s home carried out by an independent trained research assistant. Quality of life (EQ5D-5 L), capability (ICECAP-O), and stressful life events were re-assessed at follow-up. New measures included at follow-up were a 5-item Likert visual analogue scale to provide a self-assessment of mood (5 faces), and an economic assessment that included utilization of private care, need for care, purchase of mental health medication, opportunity costs related to the disease (measured by the time spent on disease-related activities), and work productivity measures. Opportunity costs measure the alternative use of time and can be monetized by considering how individuals value time (usually the monetary value of time is the individual’s work productivity measure, such as their salary or pension per unit of time).

#### Intervention costs

In addition to the information collected during the follow-up interview, we explored the possibility of extracting additional information through linkage with existing databases storing routinely collected data on patients’ use of medication, consultations, and other treatments related to their mental health. As our aim was to collect information on the costs of the intervention, we excluded ‘sunk’ costs, such as development of the depression programme’s media resources, which would not recur in practice. We also excluded the costs of the initial identification and screening of patients. We assumed that in practice NAs/CHWs would conduct PHQ-9 screening as part of their routine regular home visits with elderly patients. We also excluded costs incurred equally in both arms of the study (such as training of family doctors and nurses). The running costs of the intervention include: the equipment and support costs for the Information Technology system (IT); the costs of training NAs/CHWs in intervention delivery; intervention delivery costs; and the costs of supervising NAs/CHWs.

### The psychosocial intervention

The intervention developed is aligned with the principles of collaborative and stepped-care, and with considerable task-shifting involved. One of the main goals of the intervention is to strengthen the autonomy of the patients and highlight the role they have to play for their own improvement. Participants should be able to, slowly, turn the vicious cycle of depression into a virtuous cycle of recovering from depression. The intervention consisted of a unique blend of psychosocial techniques tailored according to the needs of each participant and with embedded support mechanisms for non-specialist health workers delivering the intervention. The main theoretical orientation is that of behavioural activation (BA) in view of its demonstrated feasibility and efficacy for the treatment of depression [37, 38]. A recent non-inferiority and cost-effectiveness study (COBRA-trial) comparing both BA and Cognitive Behavioural Therapy (CBT) for depression showed that behavioural activation can be delivered by junior mental health workers with no lesser effect and at less cost than CBT [39]. Recent meta-analyses have demonstrated its effectiveness in treating older adults with depression [37, 38], with improvement rates similar to those achieved through medication and often preferred by the elderly [18, 19]. It is a simple technique to apply and requires only a short period of professional training [40, 41]. Briefly, behavioural activation promotes the engagement in pleasant activities, which increases positive interactions with their environment. Behavioural activation is eminently suitable for delivery by non-specialists [40, 42, 43]. Furthermore, the intervention incorporates elements of psychoeducation (that is, education about depression and simple coping strategies to deal with depressive symptoms and associated problems), and relapse prevention (that is, simple strategies to remain euthymic). There is continuous monitoring of depressive symptoms with the use of the PHQ-9 depression scale and management for other chronic health problems.

**Fig. 1:**
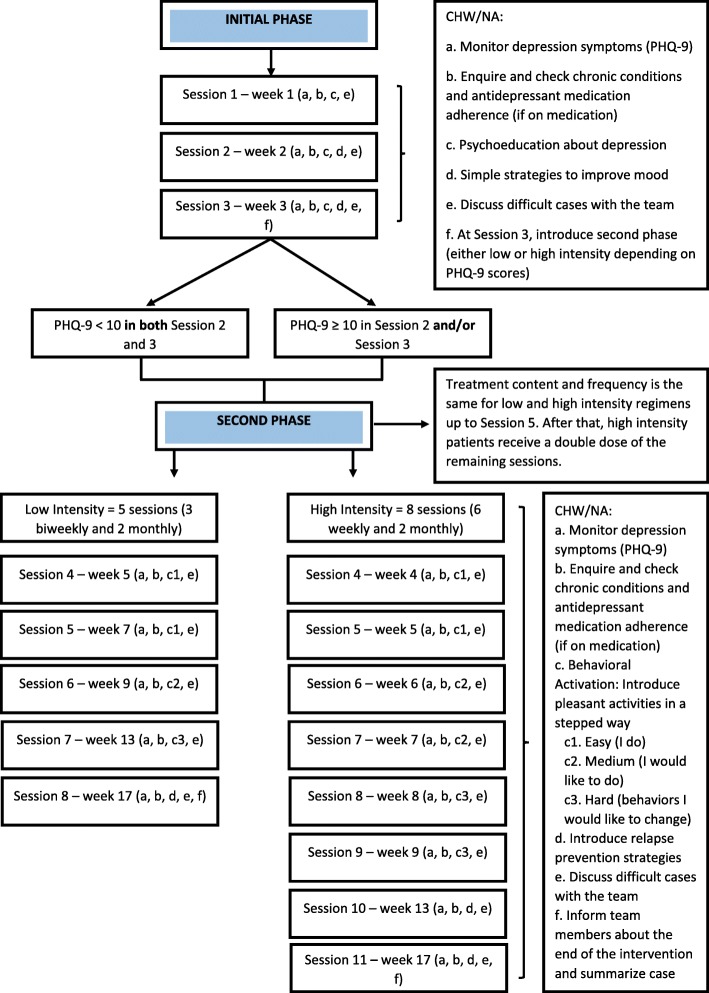
The Psychosocial Intervention Flow

The intervention itself is divided into Initial (3 weeks) and Second (14 weeks) Phases (Fig. 1). Home sessions were face to face and lasted approximately 60 min (for detailed information, see Table 3).

#### Initial phase

All participants complete the Initial Phase, which includes three weekly meetings. The goal of this phase is to provide psychoeducation about depression and develop, along with the patient, simple strategies to deal with depressive symptoms. In all sessions, NAs/CHWs measure depression symptoms and enquire about a list of common chronic physical conditions. If any chronic physical condition is present, NAs/CHWs check if these are under active control and the level of adherence to medications, if prescribed.

#### Second phase

During the Second Phase, participants access either low or high intensity regimes. If the patient has improved sufficiently after the Initial Phase (PHQ-9 < 10 in both Session 2 and 3), they proceed to the Second Phase, low intensity regime, which includes five additional meetings (3 biweekly and 2 monthly). If the patient does not improve sufficiently (PHQ-9 ≥ 10 in Session 2 and/or Session 3), they are referred to the high intensity regime that includes eight additional meetings (6 weekly and 2 monthly). The intervention lasts for 17 weeks in total, regardless of the regime. The goal of the programme’s Second Phase is to teach patients behavioural activation and relapse prevention techniques. The focus of care is thus on increasing patient involvement in pleasant activities, on reducing avoidant or excessive behaviours associated with symptoms of depression, and on strengthening the ability of the patient to identify and deal with symptoms of depression.

The intervention was designed to be delivered by NAs or CHWs. We chose these health workers to deliver the intervention because they are part of the FHT, visit homes regularly, and are involved in the care of chronic conditions. Delivery at home was selected for several reasons: older adults have difficulties travelling, an intervention at home is likely to improve adherence, the CHWs visit homes at least monthly and NAs make visits whenever it is needed, and because it provides an opportunity to assess the home environment and to contact carers, if available. Health workers were supported through a specially designed technological platform, and continuous supervision delivered by psychologists.

The technological platform contained a tablet application that included: (a) the structure of each session to guide the NA/CHW during the intervention. This structure is adapted to the specific needs of the participant as identified during the session (for example, an extra questionnaire in case of suicidality, homework tailored to problems identified during the session) and as determined by the severity of depressive symptoms in the initial phase of the intervention; (b) a function to schedule appointments with the participant, keep track of missed or moved appointments; (c) graphs with mood ratings, adherence to homework, and algorithms that activate notification to various stakeholders (such as NAs/CHWs, managers, clinical supervisor); (d) a structured approach for choosing, planning, and assessing adherence to homework; (e) an automated notification system to warn the clinic manager about the need to discuss participants who did not improve or showed high suicidal risk, the attendance of those delivering the intervention to supervision, and/or delays in delivering sessions; and (f) a function for audio recording of sessions for use in supervision if needed. The tablet application is enriched with media resources created collaboratively between media professionals and the research team. Twenty-three animated short videos were developed, highlighting all the main contents of the intervention. Three animated characters were created to portray, respectively, a person adherent to treatment, a participant with some problems with adherence, and the health care provider. The technological platform also stores participant data collected during assessments and sessions, and allows access to this dataset through tablets or computers. Web interfaces were created to allow monitoring of participant progress in the trial by the research team – for instance, inclusion of participants in the trial, start date of the intervention, number of sessions completed and follow-up date.

### Enhanced usual care

Participants included in the intervention and control arms of the pilot study received ‘enhanced usual care’, adding (1) identification of depression and (2) additional training of nurses and family doctors to the usual care. The FHT was responsible for delivering usual care for both the control and intervention clinics in the study. Briefly, usual care in clinics is carried out through consultations with nurses and/or family doctors. Whenever needed, NAs, nurses or family doctors might visit patients at home. In general, households registered with the clinic receive a monthly visit from CHWs. Face-to-face consultations at the clinic also take place, if needed. Health professionals assess each case and either initiate treatment or refer patients to more specialized health care professionals. When patients are referred to specialized care, they continue to be seen by the team simultaneously to ensure continuity of care. Regarding mental health, the clinic usually relies on support from the Family Support Team (NASF in Portuguese), which includes psychologists, nutritionists, physiotherapists, speech therapists, occupational therapists, and psychiatrists. If patients need to be seen by specialized mental health care professionals, they can be referred to psychiatrists and psychologists at the Psychosocial Care Centres (CAPS in Portuguese).

### Training

We developed treatment protocols to cover the requirements of non-specialist health workers delivering the intervention. These health workers participated in a training programme and received continuous group supervision. The training programme consisted of three full days of training delivered by two research psychologists. The training included an overview of the intervention, discussion about depression and its treatment in older adults, specific session contents, psychosocial techniques to deliver the intervention, ways to engage with patients, and how to use the technological support platform. The continuous group supervision (up to six health workers in each group) was delivered by a research psychologist and included discussion of cases and review of session contents. Initially the group supervision was weekly and then biweekly, when sessions became less frequent.

Nurses and family doctors in both arms received a brief training session before the pilot study started. A psychiatrist and a member of the research group delivered the training in each clinic. It consisted of a 90-min lecture about depression and depression care for elderly patients, medication management, followed by an approximately one-hour discussion about the pilot study protocol. In the intervention clinic, the discussion was about how cases of depression would be identified by the research team and referred to the intervention, and about the core principles of the intervention (collaborative, stepped-care, and task-shifting). It was also announced that an on-call psychiatrist would be available to provide advice about the treatment of patients included in the pilot study, and how they could contact this psychiatrist (initially by email). In the control clinic, we explained that after identification of cases of depression by the research team, a list with the names of all patients with depression from their clinic included in the study would be sent to the clinic’s manager, who would be responsible for informing the team. After this, the team professionals would be responsible for the management of these cases as in usual care.

### Data analysis

For the purposes of the pilot study, the analyses utilized just descriptive statistics such as frequencies, proportions/percentages, means and standard deviations (SD). Given our aims and that numbers in this pilot study were too small for reliable inferences, between-group comparisons of outcomes are not appropriate. This manuscript adheres to CONSORT guidelines.

## Results

### Aim 1. To assess the feasibility of recruitment, assessments, and random selection of participants

**Fig. 2:**
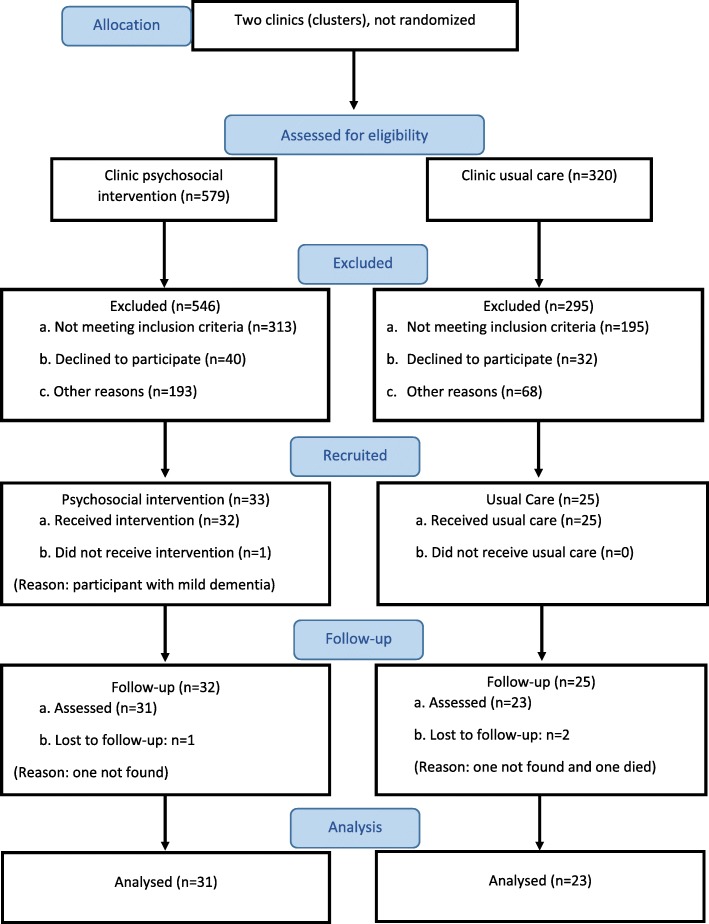
CONSORT flow diagram of the pilot study

In the intervention arm, CHWs for the seven FHTs provided details of 2020 individuals. To reflect our plans for the definitive trial given the sample size requirements detailed below, we then sampled at random 579 individuals (28.7%) for potential inclusion (Fig. 2). Of these 579, PHQ-9 scores were obtained for 353 (61.0%), of whom 40 (11.0%) scored at least 10. Of these 40, 33 (82.0%) consented to enter the pilot – which corresponds to a yield of 5.7% of the original 579 sampled. In the control arm, CHWs of the three FHTs provided details for 1482 individuals, we sampled at random 320 (21.6%), and PHQ-9 scores were obtained for 223 (69.7%). Among the 28 (12.6%) participants with PHQ-9 ≥ 10, 25 (89.0%) consented to participate in the study, with a resulting yield of 25/320 (7.8%). The age and sex distributions between those sampled and recruited were similar in both arms; however, in the intervention arm there was a marginal over-representation of females and individuals 70 or more years old amongst those recruited (data not shown). Overall, 58 out of 68 eligible individuals (85.0%) consented, producing a yield of 58 (6.5%) out of 899 sampled across the two clinics. Recruitment started in October 2015 and ended in December 2015.

The characteristics of recruited individuals in both arms are given in Table 1. There were very few missing values for any of the variables among those sampled for inclusion. It is also worth noting that, while the baseline mean PHQ-9 score was slightly lower in the control than the intervention clinic (13.9 and 15.5 respectively), the standard deviations were very similar between these two clinics (Table 1).

**Table 1:** Characteristics of the individuals in both arms of the pilot

Variables	Categories	Control (*n* = 25)	Intervention (*n* = 33)	Total (*N* = 58)
Age	60–64	24%	9%	15%
	65–69	16%	27%	22%
	> = 70	60%	64%	62%
Sex	Female	80%	73%	76%
	Male	20%	27%	24%
Education (in years)	< 5	64%	73%	69%
	> = 5	36%	27%	31%
Personal income^a^ (Brazilian minimum wage units, US$288.30) ^b^	<=2	84%	94%	89%
	> 2	4%	6%	5%
PHQ-9 (baseline)	Mean (*SD*)	13.9 (3.7)	15.5 (3.5)	14.8 (3.6)

^a^3 missing cases in the control group

^b^Exchange rate US$/Real (1US$ = R$3,25)

In terms of the follow-up, 23 out of 25 (92%) in the control, and 31 out of 33 (94%) in the intervention arm provided a score on depression (PHQ-9) at 26-weeks post-recruitment (Table 2). It can be seen that (albeit with small numbers) for all measures (PHQ-9, EQ-5D-5 L, and ICECAP-O), the intervention group commences with poorer results and ends with similar or slightly better outcomes in comparison with the control group (Table 2). As stated earlier, formal comparisons are not appropriate given the aims and the design of the pilot study (including the small numbers), but this is at least encouraging and the general lessons that were learnt from these data will be covered in the discussion. Other characteristics of participants, such as physical incapacity, social support, stressful life events, use of alcohol and tobacco were successfully collected in both arms of the study (results not shown), supporting the feasibility of collecting this type of data with this population. We did not identify any harm or unintended effects in participants of both arms of the pilot trial.

**Table 2:** Patient Health Questionnaire-9 (PHQ-9), European Quality of Life 5 Dimensions-5 levels version (EQ-5D-5 L), and Investigating Choice Experiments for the Preferences of Older People-CAPability (ICECAP-O) [Mean (SD)] at baseline and follow-up for the control and intervention arms

Measure	Time Point	Control		Intervention		Total	
		*N*	*M* (*SD*)	*N*	*M* (*SD*)	*N*	*M* (*SD*)
PHQ-9	Baseline	25	13.9 (3.7)	33	15.5 (3.5)	58	14.8 (3.6)
	Follow-up	23	12.3 (3.7)	31	3.8 (3.9)	54	7.4 (5.7)
EQ-5D-5 L^a^	Baseline	25	0.8081 (0.13)	33	0.7127 (0.16)	58	0.7539 (0.15)
	Follow-up	23	0.8202 (0.13)	31	0.8121 (0.14)	54	0.8156 (0.14)
ICECAP-O	Baseline	3^b^	0.6631 (0.14)	33	0.5564 (0.18)	36	0.5653 (0.18)
	Follow-up	23	0.7067 (0.15)	30	0.7328 (0.16)	53	0.7214 (0.15)

^a^EQ-5D-5 L and ICECAP-O higher scores represent better outcomes

^b^The ICECAP-O was administered only to a few participants in the control group at baseline because we were initially uncertain of its feasibility for people with low levels of literacy. After testing the questionnaire and concluding that participants could provide a valid response to the ICECAP-O, we collected baseline information with 3 control and all intervention participants

### Aim 2. To obtain an estimate of the outcome variance and recruitment/retention rates to inform sample size calculations for the definitive RCT

A sample size calculation based on the information collected in this pilot study (the intra-cluster correlation coefficient of 0.03 used to calculate the sample size was based on our previous study^43^ and on the literature) showed that the definitive RCT would require 20 clinics (clusters), with the inclusion of 1440 depressed older adults to detect a 15-percentage point difference in the primary outcome with 86.5% power and 15% attrition. If the 7% attrition rate found in the pilot pertained in the definitive trial, then we would have a power of approximately 90% to detect the target difference of 15%.

During this pilot study, we identified that there are on average 400 individuals in the eligible age range registered with each FHT (data not shown), and therefore 40 individuals potentially eligible (assuming 10% prevalence of depression). We are likely to have 24 individuals per team once the entry criteria are applied (approximately 6% of the total). The experience from the pilot indicates that we should work with four teams per clinic, three CHWs per team, and that each CHW can manage at least three participants at any given time. For this reason, we will only recruit 18 of these 24 individuals and plan to conduct the RCT in two waves. In each wave, a total of 36 individuals per clinic (cluster) will be included.

### Aim 3. To evaluate the feasibility of delivering the psychosocial intervention, and to compare the performance of community health workers (CHWs) and nurse assistants (NAs) delivering the intervention

**Fig. 3:**
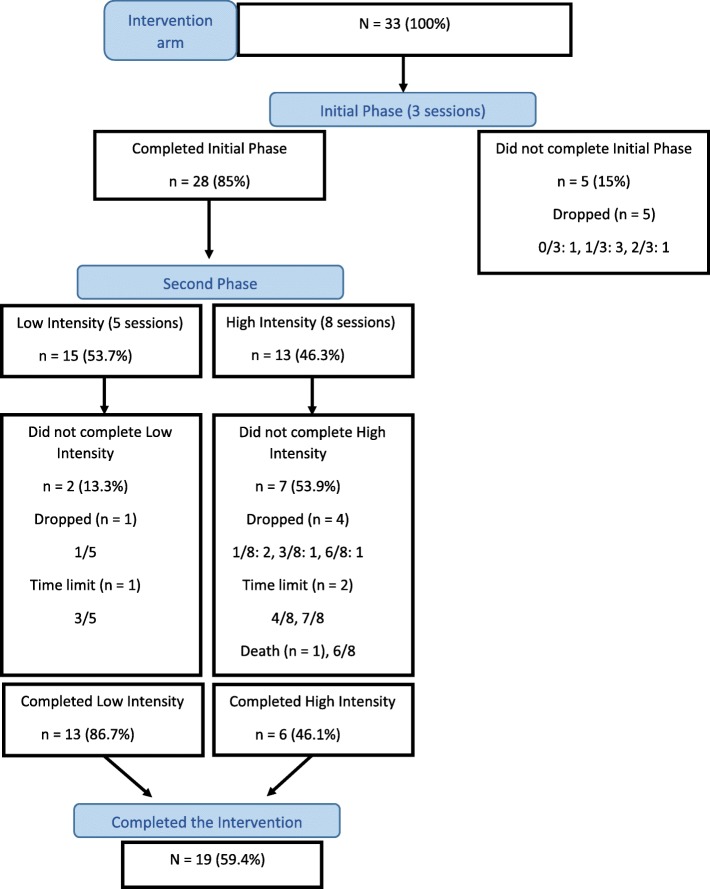
Flow diagram of the intervention arm

Regarding the process of delivering the intervention, Fig. 3 shows that of the 33 individuals who consented, five withdrew from the intervention during the initial phase (the first three sessions). Of the 28 who proceeded to the second phase, 15 (54.0%) followed the low intensity and 13 the high intensity route. Of these two groups, 13 (87.0%) and six (46.0%) completed all of the intended sessions respectively. However, three individuals did not complete the session simply due to the slightly curtailed time available for the follow-up in the pilot study – the time limit for completing the intervention was 24 weeks after patients were assigned to health workers (Fig. 3).

Three NAs (from three FHTs) and eight CHWs (from four FHTs) delivered the intervention. Comparing the NA-managed participants with those managed by CHWs, the completion rate was higher for the former (8/9, 88.9%, and 11/24, 45.8%) but numbers were very small and all but one of the high intensity participants were in the CHWs’ group. Considering the low intensity group only, the percentages completing were 100% (7/7) and 75% (6/8) for the NA and CHW group respectively. While these involve very small numbers, no noticeable differences between the health workers’ groups are suggested from these figures.

Approximately 94% of the CHWs and NAs attended each of the 3 days training. The group supervision was also well attended by CHWs and NAs (approximately 85% attendance on each day). As the supervision progressed, we found that CHWs/NAs needed technical support between sessions, mostly due to issues regarding the tablet application. Therefore, the supervisors created a WhatsApp network to deal with these issues. The training programmes conducted in the intervention and control clinics were well attended by nurses and family doctors. The psychiatrist on-call was never contacted by the nurses or family doctors from the intervention clinic, despite repeated reminders sent to the teams after the initial training that this resource was available.

### Aim 4. To assess the feasibility of collecting information on use of resources, including costs associated with intervention delivery, and health data from existing health system databases, to conduct an economic evaluation during the definitive trial

Regarding the feasibility of collecting health data from health system databases, we found that while it was theoretically possible to link to routinely collected electronic health systems data, we were unable to implement an automated method of extracting data from these systems. Data extracted from the health system databases suggested that there were no substantial differences in terms of prescribing antidepressant medication in the intervention and control groups during the study.

The IT equipment costs during the pilot involved a Personal Computer server hosted within the University of São Paulo service. For a larger roll out of the system, the IT resources would include a more powerful server including redundancy, data backup, power supply and software licensing. In addition, tablets, SIM cards and keyboards are needed for the health workers delivering the intervention. The cost of trainee and trainer time includes a three-day training course for health workers before the start of the psychosocial intervention, and supervisory group sessions during the intervention period. All of these costs are fixed or semi-fixed costs that do not increase linearly with the number of patients treated. If only CHWs delivered the intervention, the intervention cost per patient (excluding the fixed and semi-fixed costs described above) would be approximately US$25.22 in the low intensity group and US$34.68 in the high intensity group (Table 3). If NAs delivered the intervention, these costs would be 65% higher.

**Table 3:** Cost of the psychosocial intervention per patient

	Low intensity	High intensity
Intervention		
Average duration of session (minutes)	52	52
Number of sessions	8	11
CHW wage per month (including tax and benefits)^a^	US$ 640.64	US 640.64
CHW days worked per month	22	22
CHW hours worked per day	8	8
CHW wage per hour	US$ 3.64	US$ 3.64
Cost of intervention (if all sessions attended) per patient	US$ 25.22	US$ 34.68

^a^Exchange rate US$/Real (1US$ = R$3.25)

## Discussion

This pilot study aimed to assess the feasibility of undertaking a definitive RCT of a psychosocial intervention targeting depression improvement among older adults. Low levels of refusal at recruitment, few exclusions when applying our exclusion criteria, high levels of consent (over 80%) among eligible participants in both arms, and low levels of attrition at follow-up, confirms that it is feasible to conduct the definitive RCT, and that the trial is likely to provide statistically precise information. Task-shifting proved to be feasible, including delivery through the home visits by CHWs.

The pilot did identify difficulties in contacting potentially eligible individuals. We noticed that the lists provided by the CHWs were out-of-date – specifically, they included individuals who were not contactable because they had moved to another area. Some eligible participants were not contacted because they were not at home when the research team tried to contact them. For the definitive trial, the research team will obtain the lists of patients directly from the clinic electronic systems and hence the overall number of older adults registered with the clinic will be much closer to the real total and the contact data will be much more up-to-date. Additionally, in the definitive RCT, we are planning to carry out two waves of recruitment so that those older adults uncontactable in the first wave might be reached and included in the second wave.

Ideally, allocation of participants should consider the individual level of depression at baseline, as its severity is known to be a good predictor of depression recovery [44–46]; this is usually achieved by stratification by a summary measure at the cluster level. Such data will not be available in advance for a definitive trial. However, we will collect individual data on severity of depression at baseline as well as follow-up and hence we will be able to adjust for the former in the relevant (primary) analysis – mainly to maximize power in the context of a cluster randomized trial [47]. Rather than restricting the allocation according to baseline depression in the definitive RCT, we will use available sociodemographic data to make sure the two groups of clusters (clinics) are as balanced as possible. We will utilise census data on educational levels (of the head of the household) for the relevant census district to stratify the (cluster) randomisation and hence avoid the imbalance on some socio-demographic variables that was arguably inevitable in this small pilot. In the definitive RCT, we aim to include 20 clinics, 72 depressed older adults in each of the two groups of clinics, giving a total of 1440 participants.

Any difference observed in the levels of the primary (PHQ-9) and other outcomes was not an important issue in the pilot study given that the purpose was not to make between arm comparisons, but to learn about feasibility of intervention and procedures. It is nonetheless worth noting that, in the pilot study, the mean PHQ-9 at follow-up in the intervention clinic was very much lower than at baseline. This observation indicates that the intervention is not only feasible but is also promising in terms of its effectiveness albeit in the context of a small pilot study where many of the key procedures were being developed and assessed.

Overall, out of the 33 participants who consented within the intervention arm of the pilot study, 28 (85%) completed the Initial Phase of the intervention (3 sessions), 19 (59.6%) completed all sessions of the intervention (8 or 11 sessions) and only one participant withdrew from the intervention before the first session, indicating good adherence to the intervention. Although the focus of the intervention is the treatment of depression, we observed during the pilot study that asking patients about other chronic conditions increased adherence to the intervention, possibly because patients felt that their health problems were being treated comprehensively.

Task-shifting challenges are not only related to patients’ acceptance of non-specialist advice, but also the ability of CHWs to adjust to their new role. To support the delivery of the intervention, we developed a tablet application with the structure of the sessions and considerable media resources available to support the activities carried out during sessions. Another challenge was related to collaborative care (that is, discussing cases with the health team). The knowledge acquired during sessions, the supervision, and the notification system embedded within the app empowered health workers to overcome usual communication barriers with more specialized professionals. Some health workers saw patients who were registered with the clinic but with another health worker. Health professionals and patients did not object to this. Our findings showed that the CHWs and NAs were well supported by health teams in so far as this programme is concerned. Lastly, bearing in mind that there do not appear to be substantial differences in delivery between CHWs and NAs, the lower costs associated with CHWs and the considerably lower disruption to the provision of other services in the clinics, suggest that CHWs are a good choice to deliver the intervention.

The pilot study suggested modifications to the training programme conducted with nurses and family doctors of the control and intervention clinics. Professionals working in the Family Health Strategy received training on how to treat and manage patients with depression, but we found no noticeable differences in this respect across clinics. For this reason, we decided to exclude this aspect of the training, as it would add extra costs to the programme with potentially little benefit. Also, as the on-call psychiatrist available for the intervention clinic was never contacted, we also decided that it was unnecessary. However, we did observe during the pilot study that the training given to the nurses and doctors from the intervention clinic seemed to have improved communication within the family health team and led to obtaining a swifter response from family doctors when there was a need to discuss patients. As a consequence, we decided to train all team members (nursing assistants, nurses, family doctors and the community health workers) from the intervention arm on improving team communication, an essential aspect of collaborative care, and not only with nurses and family doctors.

Whilst we are investigating if an automated method of extracting data from health system databases is technically feasible for the definitive RCT, we will consider whether it would be more feasible to collect key items of health care use (such as antidepressant medications) directly from patients. An economic evaluation alongside the definitive trial could estimate the trade-off between intervention costs, any subsequent increases or decreases in healthcare costs and improved outcomes measured by the EQ-5D-5 L, ICECAP-O, and PHQ-9. The cost estimations reported in this paper suggest that the intervention is affordable, but they are preliminary as the number of participants is small. In a definitive RCT, cost data might be expanded, for instance to include the indirect costs of depression due to time off work or usual activities.

This pilot study provided evidence in favour of the feasibility of a definitive RCT of this psychosocial intervention targeting depression improvement in older adults. In Brazil, as in other LMICs, there is a gap with respect to integrating mental health into primary care services. This gap is even more pronounced when it comes to depression in late life. Therefore, a pragmatic trial focusing on improving identification and treatment of depression in older adults in primary care is urgently needed. A positive outcome may constitute a timely contribution to evidence-based treatment options for depressed older adults and reduce health costs and dependency on specialized mental health resources, a problem encountered in most LMICs. This study is registered with Registro Brasileiro de Ensaios Clínicos (ReBEC), number RBR-5nf6wd.

## Conclusions

In this pilot study we assessed the feasibility of recruitment, assessments, randomization as well as other procedures to conduct a cluster randomized controlled trial of an intervention to treat depressed elderly people living in socio-economically deprived areas of Sao Paulo. Recruitment, assessments, randomization as well as most other procedures (Aim 1) were found to be highly feasible within this setting. We were also able to ascertain some essential parameters to estimate the sample size as well as retention rates (Aim 2), both of which proved to be auspicious. We also found it feasible to deliver the intervention in primary care clinics and the two types of health workers that were trained (NAs and CHWs) could equally deliver the intervention (Aim 3). We investigated the possibility of collecting cost-related information associated with the intervention delivery, and health data from existing health system databases (Aim 4). Our preliminary results suggest that routinely collected data is still not sufficiently reliable or complete to conduct a proper cost-effectiveness analysis. Thus, we will collect most of the cost data as part of the research component when we start the fully powered RCT. In Brazil, as in other LMICs, there is a gap with respect to integrating mental health into primary care services. This gap is even more pronounced when it comes to depression in late life. Therefore, a pragmatic trial focusing on improving identification and treatment of depression in older adults in primary care is urgently needed.

## Data Availability

The datasets used and/or analysed during the current study are available from the corresponding author on reasonable request.

## Abbreviations

AUDIT: Alcohol Use Disorders Identification Test
CAPS in Portuguese: Psychosocial Care Centres
CHW: Community Health Worker
CONSORT: Consolidated Standards of Reporting Trials
EQ-5D-5 L: European Quality of Life 5 Dimensions-5 levels version
FHS: Family Health Strategy
ICECAP-O: Investigating Choice Experiments for the Preferences of Older People-CAPability
IT: Information Technology System
LMIC: Low-Middle-Income Countries
NAs: Nurse Assistant
NASF in Portuguese: Family Support Team
PC: Registro Brasileiro de Ensaios Clínicos (ReBEC)
PHQ-9: Patient Health Questionnaire-9
RCT: Randomized controlled trial
SD: Standard Deviation
